# Identification and Characterization of the Designer Opioid *N*‐Pyrrolidino Fluetonitazene in Nasal Spray

**DOI:** 10.1002/dta.70074

**Published:** 2026-04-15

**Authors:** Fabian Picht, Valentin Cepus, Rona Hohlfeld, Julian Klingbeil, Marco Weber

**Affiliations:** ^1^ Institute of Legal Medicine University of Halle‐Wittenberg Halle (Saale) Germany; ^2^ Department of Engineering and Natural Sciences University of Applied Sciences Merseburg Merseburg Germany; ^3^ Department of Neurology University of Halle‐Wittenberg Halle (Saale) Germany

**Keywords:** nasal spray, new psychoactive substance, nitazene

## Abstract

Detection of drugs in non‐biological samples serves as a fundamental basis for identifying emerging trends in the field of new psychoactive substances and provides valuable information for optimizing analytical methods within the field of chemical analysis. Nitazenes belong to this group of compounds and have been reported to cause poisonings. *N*‐pyrrolidino fluetonitazene, also known as fluetonitazepyne (2‐(4‐(2‐fluoroethoxy)benzyl)‐5‐nitro‐1‐(2‐(pyrrolidin‐1‐yl)ethyl)‐1*H*‐benzo[*d*]imidazole), is another emerging synthetic opioid that may contribute to the challenges faced globally. This article describes the identification and characterization of *N*‐pyrrolidino fluetonitazene in a rarely reported medium for opioids: nasal spray. The sample investigated here was collected from a patient treated at the University Medicine Halle following first‐time recreational use of a nasal spray and was submitted in an amber plastic bottle containing a small volume of a yellowish viscous liquid. The analytical techniques used for identifying this compound included gas chromatography–mass spectrometry, liquid chromatography–quadrupole time‐of‐flight mass spectrometry, nuclear magnetic resonance spectroscopy, and Fourier transform infrared spectroscopy.

## Graphical abstract

*N*‐Pyrrolidino fluetonitazene, a synthetic opioid, was identified and characterized in a nasal spray using GC‐MS, LC‐QTOF‐MS, NMR and FT‐IR.

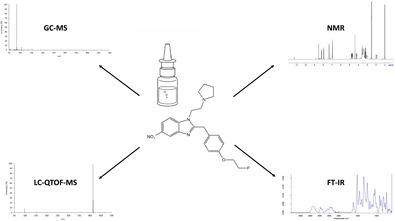

## 1 Introduction

During the last decade, synthetic opioids, alongside other new psychoactive substances, have entered the drug market [1]. A novel group of these compounds are 2‐benzyl benzimidazoles, so‐called nitazenes. Characterized by a benzimidazole ring with an ethylamine and a benzyl group, further structural modifications are possible and have already been reported [1, 2, 3, 4]. Initially, these substances were synthesized in the 1950s and investigated for their analgesic properties, but therapeutic use has never been approved [5]. However, first detected in 2019 and represented by isonitazene and leading to hundreds of fatalities worldwide [6, 7], numerous analogues have emerged since then [8]. Despite the risks associated with the illicit use, current data indicate an increasing prevalence of use of this substance class [1]. The effects, similar to other opioids, are also accompanied by not only a much higher but also a substitution‐dependent potency [9, 10]. For example, etonitazene was found to be 10 times more potent than fentanyl [9, 10]. However, the emergence of nitazenes is primarily driven by legislative changes, such as Chinas's 2024 controls on 10 nitazene opioids, which shifted markets towards novel derivatives [1]. As nitazenes pose a significant risk to public health with new derivatives emerging persistently, the continuous adaptation of analytical methods to detect them is a necessity.

Detections of nitazenes in samples of non‐human origin have been reported for powders or tablets [4, 8, 11, 12, 13]. Nasal sprays represent an additional form among the recreational drug market, although detections are rarely reported, especially in relation to opioids. The reason for this may be that the overall sample size in nasal sprays is significantly smaller than those in seized powders or tablets or that such samples are seldom submitted for analysis. From an analytical point of view, it must additionally be taken into account that other components in the sample, such as excipients or solvents, can lead to insufficient analytical results and may necessitate additional sample preparation such as solvent removal. Despite all that, the opioid U‐47700 has been identified as the cause of a fatal intoxication and was detected in nasal spray alongside other biological samples [14]. Furthermore, butyrfentanyl, 4‐fluorobutyrfentanyl and fentanyl have also already been detected in nasal spray [15]. Cyclopropylfentanyl is reported to be the cause of an intoxication, even though the analysis of the nasal spray was not reported [16]. In addition, the identification of a substance in non‐biological samples is important as the components actually present may be different from what the user assumes to ingest and from the labelling of the product [8, 15].

Most frequently used techniques for the identification and characterization of nitazenes in this setting include gas chromatography–mass spectrometry (GC‐MS) [4, 8, 9, 12, 13], liquid chromatography–quadrupole time‐of‐flight mass spectrometry (LC‐QTOF‐MS) [8, 9, 12, 17], nuclear magnetic resonance spectroscopy (NMR) [4, 8, 9, 12, 13] and Fourier transform infrared spectroscopy (FT‐IR) [4, 8, 12, 13].

This article focuses on the identification and characterization of *N*‐pyrrolidino fluetonitazene (2‐(4‐(2‐fluoroethoxy)benzyl)‐5‐nitro‐1‐(2‐(pyrrolidin‐1‐yl)ethyl)‐1*H*‐benzo[*d*]imidazole) in a nasal spray. This substance represents another emerging member of nitazenes and has been recently detected in post‐mortem samples [18]. Unambiguous analytical identification was performed via GC‐MS, LC‐QTOF‐MS, NMR and FT‐IR and by comparison with a reference standard.

## 2 Materials and Methods

### 2.1 Chemical Reagents

All reagents used during analyses were at least of HPLC grade. For GC‐MS analysis, methanol (HPLC grade) was purchased from Merck (Darmstadt, Germany). For LC‐QTOF‐MS analysis, MS‐grade water, MS‐grade methanol, MS‐grade formic acid and MS‐grade ammonium formate were purchased from Biosolve (Valkenswaard, the Netherlands). Deuterated dimethyl sulfoxide (DMSO‐d6) was purchased from Sigma‐Aldrich and used for NMR analysis. The standard of *N*‐pyrrolidino fluetonitazene was from Cayman Chemical (Ann Arbor, MI, USA).

### 2.2 Sample

The sample was obtained from a 34‐year‐old male patient at the University Medicine Halle who had presented to the emergency department with a severe right arm paresis. He had perceived the paresis when waking up 2 days before and had since hoped in vain for a spontaneous resolution. He disclosed first‐time recreational use of the nasal spray 3 days prior to symptom onset. The sample arrived in an amber plastic bottle containing 4.09 g of a yellowish viscous liquid. Because no further information was available, it remained unclear to what extent the viscosity of the nasal spray allowed for adequate application or whether the product was further diluted before use.

### 2.3 Sample Preparation

For GC‐MS analysis, an aliquot of the sample was diluted 1:10 (v/v) with methanol and directly injected. LC‐QTOF‐MS analysis was performed after 1:100 000 (v/v) with the mobile phases (starting conditions). Because the concentration and composition of the nasal spray were unknown, the sample was subjected to solvent removal. For this purpose, the nasal spray (4.09 g) was transferred into a 100‐mL round flask placed in a vacuum bell jar equipped with a two‐step pump system consisting of a rotary vane pump and a turbomolecular pump. The solvent was evaporated by a stepwise reduction of the pressure from atmospheric pressure to ~1 Pa until a viscous yellowish residue (~23 mg) remained in the flask. Then 0.5 mg of this residue was used for IR measurements. A portion of 5 mg was transferred into an NMR tube and dissolved in 500 μL of DMSO‐d6. The standard of *N*‐pyrrolidino fluetonitazene was prepared for NMR spectroscopy likewise.

### 2.4 Instrumentation

#### 2.4.1 Gas Chromatography–Mass Spectrometry (GC‐MS)

The analysis was performed using an Agilent 7890B gas chromatograph equipped with a 5977A mass selective detector and an HP‐5MS capillary column (30 m × 0.2 mm I.D., 0.25 μm film thickness) (Waldbronn, Germany). The injector was operated in splitless mode with an injection volume of 1 μL. The inlet temperature and helium flow rate were set to 280°C and 1.5 mL/min, respectively. The oven temperature gradient programme was as follows: Hold an initial temperature of 80°C for 2 min, ramp to 280°C at 15°C/min and hold for 15 min, then ramp to 310°C at 5°C/min and finally hold for 25 min. The source and transfer line temperatures were 280°C and 300°C. The mass spectrometer was operated in positive electron ionization mode at 70 eV. The acquisition range was set to m/z 50–500 using scan mode. Automation was achieved using MassHunter data acquisition and data handling software.

#### 2.4.2 LC‐QTOF‐MS

High‐resolution accurate mass (HRAM) analyses were carried out with a Shimadzu Nexera HPLC system coupled to a Shimadzu LCMS‐9030 quadrupole time‐of‐flight mass spectrometer (Kyoto, Japan). Chromatographic separation was achieved using a Shim‐pack Velox Biphenyl (2.1 × 100 mm; 2.7 μm) column (Shimadzu, Kyoto, Japan) maintained at 40°C with the following flow gradient: 0.3 mL/min (0–11 min), 0.5 mL/min (11–12 min), 0.3 mL/min (12–12.5 min). The mobile phases consisted of water (A) and methanol (B) with 2 mM ammonium formate and 0.002% formic acid. The elution programme was as follows: 5% B (0–1 min), 5%–40% B (1–2 min), 40%–100% B (2–10.5 min, hold for 1 min), 5% B (11.5–12 min, hold for 0.5 min). The total run time was 12.5 min. The injection volume was 1 μL. High‐resolution data were acquired in positive electrospray ionization (ESI) mode using data independent acquisition (DIA). The precursor MS acquisition scan range was m/z 100–1000. For product MS, 20 consecutive DIA‐MS/MS scans were created isolating 20 Da ion width starting from m/z 100–120 through to 480–500 acquiring a measurement mass range of m/z 50–500, each MS/MS scan lasting 25 msec. In the MS/MS experiment, collision‐induced dissociation (CID) was carried out with argon gas, and the collision energy was set between 5 and 55 eV. The source voltage was 1.5 kV, with a nebulizing gas flow of 3 L/min, heater gas flow of 10 L/min, interface temperature of 300°C, drying gas flow of 10 L/min, desolvation line temperature of 250°C and heater block temperature of 400°C. All drying gases used were nitrogen. All data were acquired using external mass calibration with sodium iodide. Data processing was achieved using LabSolutions and the LabSolutionsInsight Explore software (Shimadzu, Kyoto, Japan).

#### 2.4.3 Nuclear Magnetic Resonance (NMR) Spectroscopy

One‐ and two‐dimensional ^1^H and ^13^C NMR spectra were acquired in DMSO‐d6 at 298 K on a Bruker Avance III 400 spectrometer (400.23 MHz ^1^H, 100.65 MHz ^13^C). Chemical shifts (δ) are reported in parts per million with ^1^H shifts referenced to the residual solvent peaks (DMSO‐d6: ^1^H δ 2.50) and ^13^C shifts referenced to the solvent peak (DMSO‐d6: ^13^C δ 39.50). Coupling constants (*J*) are reported in Hertz (Hz). Standard abbreviations indicating multiplicity were used as follows: m, multiplet; t, triplet; d, doublet; and s, singlet.

#### 2.4.4 FT‐IR Spectroscopy

The IR spectra were acquired on an FT‐IR spectrometer Vertex 70 by Bruker and a platinum diamond ATR cell in the spectral range of 4000–400 cm^−1^ and with a resolution of 4 cm^−1^. The signals were processed and labelled using the instrument software OPUS. Data manipulations were automatic atmospheric compensation and rubber‐band baseline correction.

## 3 Results and Discussion

### 3.1 GC‐MS

**FIGURE 1:**
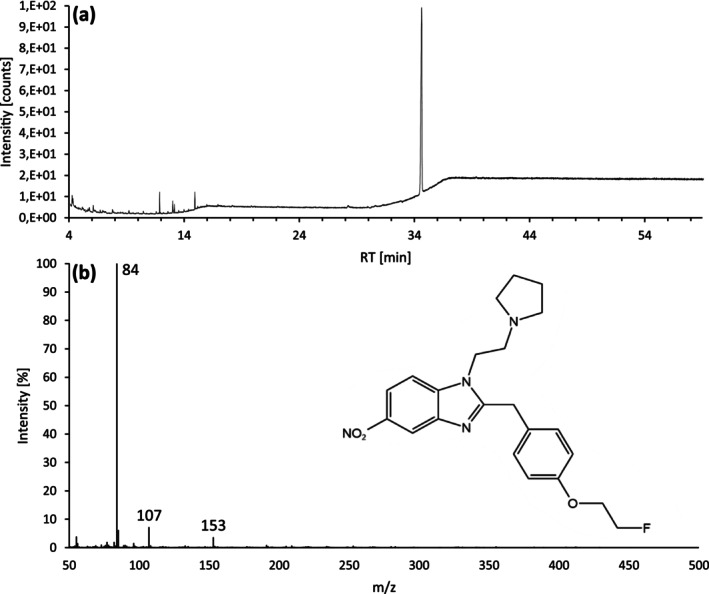
Total ion chromatogram from GC‐MS analysis of the nasal spray (a) and the corresponding spectrum (b).

GC‐MS analysis demonstrated the presence of one single peak (Figure 1a), eluting at retention time (RT) 34.06 min. The EI mass spectrum (Figure 1b) showed a base peak at m/z 84, consistent with a pyrrolidine‐type iminium ion, in addition to m/z 153 and 107, suggested to reflect a benzylic cleavage and formation of the corresponding tropylium ion. This in turn experienced a neutral loss of fluoroethene. Examples of ions at m/z 84 and 107 have also been reported for *N*‐pyrrolidino etonitazene and other derivatives [12, 19]. RT and mass spectral comparisons were eventually possible when the N‐pyrrolidino fluetonitazene reference material became available.

### 3.2 LC‐QTOF‐MS

**FIGURE 2:**
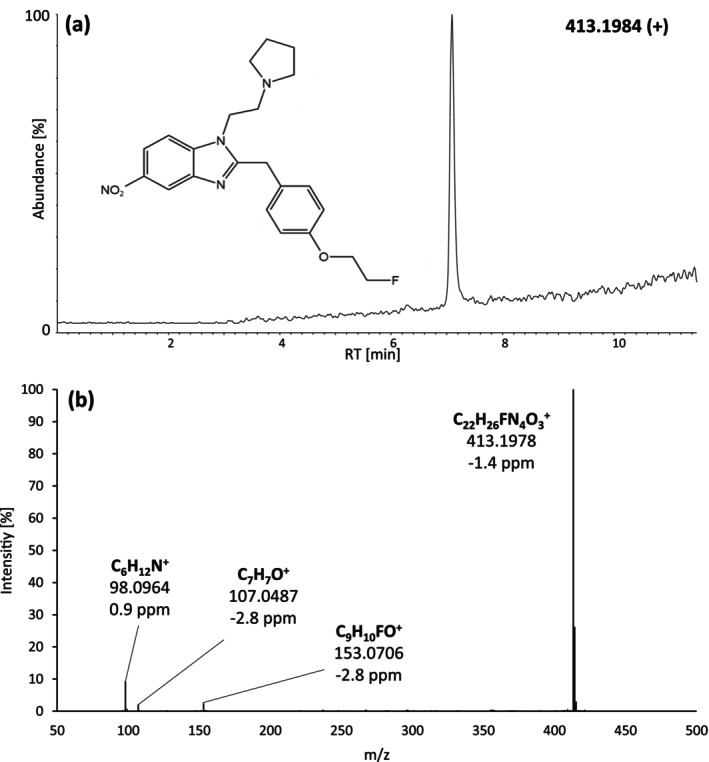
High‐resolution total ion chromatogram of the nasal spray (a) and the fragment ion spectrum (b).

The full scan data (Figure 2) revealed a [M + H]^+^ signal at m/z 413.1978 with RT 7.9 min consistent with *N*‐pyrrolidino fluetonitazene and a molecular formula of C_22_H_25_FN_4_O_3_ (theoretical m/z 413.1984, ∆ = −1.4 ppm). The most prominent product ions observed were m/z 98.0964, corresponding to the pyrrolidine moiety previously reported for *N*‐pyrrolidino fluetonitazene [18], followed by m/z 107.0487 and 153.0706. Here, a post comparison using the reference material could also confirm the initial results regarding RT and HRMS spectra.

To additionally exclude the presence of other possible nitazene candidates, the sample was screened against a library prepared in‐house. The following analytes were included: 3′‐methoxy metodesnitazene (C_22_H_29_N_3_O_2_), 4′‐hydroxy nitazene/*N*‐desethyl etonitazene (C_20_H_24_N_4_O_3_), 5‐methyl etodesnitazene/isotodesnitazene/protodesnitazene (C_23_H_31_N_3_O), 5‐methyl metodesnitazene/etodesnitazene (C_22_H_29_N_3_O), 5‐trifluoromethyl isotonitazene (C_24_H_30_F_3_N_3_O), butonitazene/iso‐butonitazene/sec‐butonitazene (C_24_H_32_N_4_O_3_), clonitazene (C_20_H_23_ClN_4_O_2_), ethylene etonitazene/isotonitazene/protonitazene (C_23_H_30_N_4_O_3_), flunitazene (C_20_H_23_FN_4_O_2_), menitazene (C_21_H_26_N_4_O_2_), methylenedioxynitazene/etonitazene (C_21_H_24_N_4_O_4_), metodesnitazene (C_21_H_27_N_3_O), metonitazene / *N*‐desethyl isotonitazene / *N*‐desethyl protonitazene (C_21_H_26_N_4_O_3_), *N*‐desethyl metonitazene (C_19_H_22_N_4_O_3_), nitazene (C_20_H_24_N_4_O_2_), *N*‐piperidinyl etonitazene/*N*‐pyrrolidino isotonitazene/*N*‐pyrrolidino protonitazene (C_23_H_28_N_4_O_3_), *N*‐piperidinyl isotonitazene/*N*‐piperidinyl protonitazene (C_24_H_30_N_4_O_3_), *N*‐piperidinyl metonitazene/*N*‐pyrrolidino etonitazene/propylnitazene (C_22_H_26_N_4_O_3_), *N*‐pyrrolidino etodesnitazene (C_22_H_27_N_3_O), *N*‐pyrrolidino metodesnitazene (C_21_H_25_N_3_O) and *N*‐pyrrolidino metonitazene/5‐aminoisotonitazene (C_21_H_24_N_4_O_3_). However, no further positive finding was given. Additional library queries for other relevant substances did not yield any matches (Shimadzu HRAM LCMS ForTox and MMHW LC‐HR‐MS/MS Drugs and Poisons).

### 3.3 NMR

The NMR spectroscopic data of the reference *N*‐pyrrolidino fluetonitazene were used to confirm the structure of the nasal spray sample by a combination of ^1^H and ^13^C spectra as well as a DEPT‐135, ^1^H‐^1^H‐COSY and HSQC experiment (Figures S1–S8). Assignments of all chemical shifts are summarized in Table 1. Combining the recorded data, 18 chemical equivalent carbon atoms and 12 proton signals were identified. Assignment for proton‐coupled ^13^C signals were determined using HSQC ^1^H–^13^C cross‐peak analysis. Additional peaks related to the citric acid/citrate counterion of *N*‐pyrrolidino fluetonitazene reference were observed at 175.49, 171.21, 71.86 and 43.33 ppm in the ^13^C NMR spectra. The presence of the fluoroethoxy group exhibited characteristic splitting patterns of the ^1^H and ^13^C nuclei near fluorine due to the spin–spin coupling. The ^13^CH_2_F at 82.13 ppm and the signal of the neighbouring O^13^CH_2_ group at 67.04 ppm each show a doublet due to the coupling with the fluorine atom (^1^ *J*_CF_ = 166.9 Hz and ^2^ *J*_CF_ = 18.9 Hz).

**TABLE 1:** ^1^H and ^13^C nuclear magnetic resonance data for *N‐*pyrrolidino fluetonitazene.

Position	Nasal spray sample		Reference	
	^1^H (δ/ppm)	^13^C (δ/ppm)	^1^H (δ/ppm)	^13^C (δ/ppm)
2	—	158.25	—	158.29
3a	—	141.54	—	141.45
4	8.49 (d, 1H, *J* = 2.2 Hz)	114.81	8.47 (d, 1H, *J* = 2.2 Hz)	114.68
5	—	142.96	—	142.66
6	8.20 (dd, 1H, *J* = 8.9 Hz, 2.2 Hz)	117.79	8.16 (dd, 1H, *J* = 8.9 Hz, 2.2 Hz)	117.61
7	7.94 (d, 1H, *J* = 8.9 Hz)	110.89	7.77 (d, 1H, *J* = 9.0 Hz)	110.80
7a	—	139.33	—	139.72
CH_2_	4.39 (s, 2H)	32.00	4.34 (s, 2H)	32.20
1′	—	128.08	—	128.30
2′, 6′	7.34 (d, 2H, *J* = 8.7 Hz)	130.22	7.24 (d, 2H, *J* = 8.7 Hz)	130.01
3′, 5′	6.95 (d, 2H, *J* = 8.7 Hz)	114.68	6.94 (d, 2H, *J* = 8.7 Hz)	114.68
4′	—	157.13	—	157.07
OCH_2_	4.25–4.18 (m, 2H)	67.03 (d, ^2^ *J*_CF_ = 18.7 Hz)	4.24–4.17 (m, 2H)	67.04 (d, ^2^ *J*_CF_ = 18.9 Hz)
CH_2_F	4.79–4.67 (m, 2H)	82.14 (d, ^1^ *J*_CF_ = 166.5 Hz)	4.78–4.66 (m, 2H)	82.13 (d, ^1^ *J*_CF_ = 166.9 Hz)
1″	4.72 (t, 2H, *J* = 7.8 Hz)	39.29 Overlapping with DMSO	4.42 (t, 2H, *J* = 6.9 Hz)	Overlapping with DMSO
2″	3.40 (m)	51.09	2.66 (m)	53.81
2″, 5″	3.55–3.02 (m, 4H)	53.13	2.84 (m)	53.81
3″, 4″	2.00 and 1.89 (m, 4H)	22.76	1.72 (m, 4H)	23.00
Citric acid	—	—	—	175.49
Citric acid	—	—	—	171.21
Citric acid	—	—	—	71.86
Citric acid	—	—	2.70–2.60	43.33
1,2‐Propane diol	3.25–3.15	67.23	—	—
1,2‐Propane diol	3.55	67.15	—	—
1,2‐Propane diol	1.00 (d, 3H, *J* = 6.3 Hz)	19.97	—	—

**FIGURE 3:**
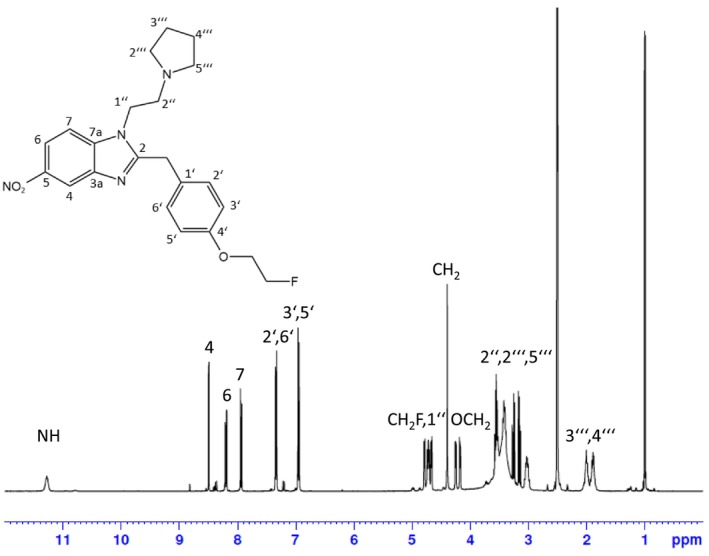
^1^H NMR spectrum of the nasal spray sample in the range of 0–12 ppm chemical shift in DMSO‐d6.

The ^1^H NMR data of the investigated sample of the nasal spray are shown in Figure 3. The main difference compared to the obtained NMR data (Figures S1–S8) of the reference *N*‐pyrrolidino fluetonitazene is the absence of the signals from citric acid/citrate counterion and the presence of remnant solvent 1,2‐propane diol, which is a component of the nasal spray formulation. Additional signals caused by incomplete evaporation of 1,2‐propane diol are observed at 19.97 ppm and around 67 ppm in the ^13^C NMR spectrum and at 1.00, 3.15, 3.25 and 3.55 ppm chemical shift in the ^1^H NMR spectrum. The sample was studied in DMSO‐d6, where the acidic N–H proton of the benzimidazolium cation typically appears as a broad singlet in the downfield region, and this signal could be observed here at a chemical shift of 11.3 ppm (Figure 3). This suggested that it may have been the HCl salt in the absence of an organic counterpart, because no NMR spectroscopic evidence could be found for the latter. Interestingly, the 1″‐methylene group showed a significant shift between sample and standard, which may have been influenced by the anion‐dependent NH proton exchange. In the region of the alkyl proton chemical shifts between 3 and 5 ppm (Figure 3), signals were tightly clustered and partially overlapped. A more detailed view is provided in Figures S3 and S8.

Although slight shifts were detected in comparison with the nasal spray sample presumably due to the influence of citric acid present in the reference sample, all signals could be assigned in a similar manner as shown before [4, 12, 20]. Thus, the identity with the reference sample *N*‐pyrrolidino fluetonitazene was confirmed by NMR spectroscopy.

### 3.4 FT‐IR Spectroscopy

**FIGURE 4:**
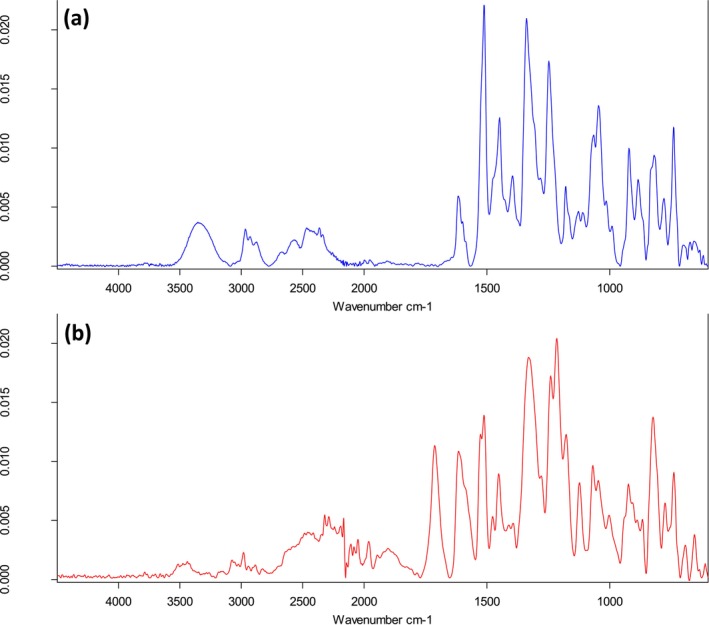
FT‐IR spectra of the nasal spray sample (a) and the reference sample *N*‐pyrrolidino fluetonitazene citrate (b) in the range of 4500–600 cm^−1^ spectral range.

The IR spectrum of the reference is shown in Figure 4b. In the upper part of the spectrum between 4000 and 2800 cm^−1^, only very weak signals could be observed. In the intermediate region between 2800 and 1900 cm^−1^, some artefacts were observed. This was partly due to the limited amount of sample, which was set aside for possible further experiments. Because this intermediate spectral region showed absorption of triple bonds and cumulated double bonds, it was decided to exclude this spectral range from the IR spectroscopic characterization, as the rest of the IR spectrum was of sufficient quality. Several repetitions of the measurement were conducted to check whether the IR spectrum could be further improved. The main reason for the artefact in the intermediate region was the insufficient function of the atmospheric compensation in the spectroscopy software used. An error message indicated incomplete convergence of the atmospheric compensation algorithm, resulting in variations in the region of CO_2_ absorption. This region was disregarded for further interpretations. In the spectral region between 1900 and 600 cm^−1^, the spectrum exhibited stable signals that were used for the interpretation and band assignment of the reference sample *N*‐pyrrolidino fluetonitazene. Because there was a large quantity of functional groups, several overlaid signals had to be addressed, and a tentative assignment following [21] was made and summarized (Table S1). The strongest bands detected could be referred to citric acid (ν(C = O) 1712 cm^−1^), the signals of citrate at (ν_as_(C‐O) 1615 cm^−1^) and at (ν_s_(C‐O) 1338 cm^−1^), the nitro group (ν_as_(N‐O) 1512 cm^−1^, ν_s_(N‐O) 1330 cm^−1^), the Schiff base of the benzimidazole ring substituent (ν(C = N) 1615 cm^−1^) and the stretching vibration of C‐F at 1240 cm^−1^.

In Figure 4a, the IR spectrum of the nasal spray sample is shown. Similar issues for interpretation can be mentioned as for the reference sample before. Likewise, the artefact region from 2800 to 1900 cm^−1^ was not used for interpretation either. However, apart from the missing carbonyl signal of the reference mentioned above at 1712 cm^−1^ caused by the absence of citric acid in this sample, some additional signals have to be taken into account due to the incomplete evaporation of the solvent propylene glycol at 3353, 2966 and 1045 cm^−1^ (Table S1). No counterion was unequivocally detected for the nasal spray sample. Because the IR spectrum exhibited a high number of overlaid signals, no further information of the counterion could be obtained. However, taking into account the previously recorded results and the high degree of similarity between the IR spectrum of the reference and the nasal spray sample, these data confirmed the presence of *N*‐pyrrolidino fluetonitazene.

## 4 Conclusion

The synthetic opioid *N*‐pyrrolidino fluetonitazene was identified in nasal spray, representing a low‐volume source for chemical analysis. The sample was obtained from a patient after presenting at the emergency department with a severe right arm paresis. Identification was based on GC‐MS, LC‐QTOF‐MS and, after solvent removal, NMR and FT‐IR spectroscopy. Given the steadily increasing number of substances entering the illegal drug market, this is relevant information as new synthetic opioids have already been a serious problem for public health due to their potency and risk of fatal intoxications. The data collected here also highlight that forensic toxicology laboratories need to keep their methods up to date in order to respond quickly to new trends and serve as a point of contact for answering subject‐related questions from law enforcement agencies, drug treatment services and healthcare professionals in hospitals. However, the current analysis was conducted on a single nasal spray product, which may limit the generalizability of the findings due to the small sample size.

## Author Contributions

**Fabian Picht:** methodology, writing – original draft, writing – review and editing. **Valentin Cepus:** methodology, writing – review and editing. **Rona Hohlfeld:** methodology, writing – review and editing. **Julian Klingbeil:** writing – review and editing. **Marco Weber:** supervision, writing – review and editing.

## Funding

The authors have nothing to report.

## Conflicts of Interest

The authors declare no conflicts of interest.

## Supporting information


**Table S1:** FT‐IR data for *N*‐pyrrolidino fluetonitazene.
**Figure S1:** dta_70074‐sup‐0001‐SupportingInformation.docx. ^1^H NMR spectrum of reference sample *N*‐pyrrolidino fluetonitazene citrate (a) and of nasal spray sample (b) in the range of 0–10 ppm chemical shift in DMSO‐d6.
**Figure S2:** dta_70074‐sup‐0001‐SupportingInformation.docx. ^1^H NMR spectrum of reference sample *N*‐pyrrolidino fluetonitazene citrate (a) and of nasal spray sample (b) in the range of 6.5–9 ppm chemical shift in DMSO‐d6.
**Figure S3:** dta_70074‐sup‐0001‐SupportingInformation.docx. ^1^H NMR spectrum of reference sample *N*‐pyrrolidino fluetonitazene citrate (a) and of nasal spray sample (b) in the range of 1.5–5 ppm chemical shift in DMSO‐d6.
**Figure S4:** dta_70074‐sup‐0001‐SupportingInformation.docx. ^1^H NMR spectrum of reference sample *N*‐pyrrolidino fluetonitazene citrate (c) and of nasal spray sample (d) in the range of 1.5–5 ppm and of reference sample *N*‐pyrrolidino fluetonitazene citrate (a) and of nasal spray sample (b) in the range of 6.5–9 ppm chemical shift with frequency notations in DMSO‐d6.
**Figure S5:** dta_70074‐sup‐0001‐SupportingInformation.docx. ^13^C NMR spectrum of reference sample *N*‐pyrrolidino fluetonitazene citrate (a) and of nasal spray sample (b) in the range of 0–200 ppm chemical shift in DMSO‐d6.
**Figure S6:** dta_70074‐sup‐0001‐SupportingInformation.docx. ^13^C NMR spectrum and DEPT‐135 spectrum of reference sample *N*‐pyrrolidino fluetonitazene citrate (a) and of nasal spray sample (b) in the range of 0–200 ppm chemical shift in DMSO‐d6.
**Figure S7:**. 2D‐HSQC NMR spectrum of reference sample *N*‐pyrrolidino fluetonitazene citrate (a) and of nasal spray sample (b) in DMSO‐d6.
**Figure S8:** dta_70074‐sup‐0001‐SupportingInformation.docx. ^1^H–^1^H‐COSY NMR spectrum of reference sample *N*‐pyrrolidino fluetonitazene citrate (a) and of nasal spray sample (b) in DMSO‐d6.

## Data Availability

The data underlying this article will be shared upon reasonable request to the corresponding author.
