# Supplementary material for: Identification and Characterization of the Designer Opioid N‐Pyrrolidino Fluetonitazene in Nasal Spray
Source: Drug Test Anal. 2026 Apr 15;18(7):802–10. doi: 10.1002/dta.70074 (PMC13327161; doi:10.1002/dta.70074)
Supplement: Supplementary file 1 — Table S1: FT‐IR data for N‐pyrrolidino fluetonitazene. Figure S1: dta_70074‐sup‐0001‐SupportingInformation.docx. 1H NMR spectrum of reference sample N‐pyrrolidino fluetonitazene citrate (a) and of nasal spray sample (b) in the range of 0–10 ppm chemical shift in DMSO‐d6. Figure S2: dta_70074‐sup‐0001‐SupportingInformation.docx. 1H NMR spectrum of reference sample N‐pyrrolidino fluetonitazene citrate (a) and of nasal spray sample (b) in the range of 6.5–9 ppm chemical shift in DMSO‐d6. Figure S3: dta_70074‐sup‐0001‐SupportingInformation.docx. 1H NMR spectrum of reference sample N‐pyrrolidino fluetonitazene citrate (a) and of nasal spray sample (b) in the range of 1.5–5 ppm chemical shift in DMSO‐d6. Figure S4: dta_70074‐sup‐0001‐SupportingInformation.docx. 1H NMR spectrum of reference sample N‐pyrrolidino fluetonitazene citrate (c) and of nasal spray sample (d) in the range of 1.5–5 ppm and of reference sample N‐pyrrolidino fluetonitazene citrate (a) and of nasal spray sample (b) in the range of 6.5–9 ppm chemical shift with frequency notations in DMSO‐d6. Figure S5: dta_70074‐sup‐0001‐SupportingInformation.docx. 13C NMR spectrum of reference sample N‐pyrrolidino fluetonitazene citrate (a) and of nasal spray sample (b) in the range of 0–200 ppm chemical shift in DMSO‐d6. Figure S6: dta_70074‐sup‐0001‐SupportingInformation.docx. 13C NMR spectrum and DEPT‐135 spectrum of reference sample N‐pyrrolidino fluetonitazene citrate (a) and of nasal spray sample (b) in the range of 0–200 ppm chemical shift in DMSO‐d6. Figure S7:. 2D‐HSQC NMR spectrum of reference sample N‐pyrrolidino fluetonitazene citrate (a) and of nasal spray sample (b) in DMSO‐d6. Figure S8: dta_70074‐sup‐0001‐SupportingInformation.docx. 1H–1H‐COSY NMR spectrum of reference sample N‐pyrrolidino fluetonitazene citrate (a) and of nasal spray sample (b) in DMSO‐d6. [file DTA-18-802-s001.docx]

**Supporting Information for**

**Identification and characterization of the designer opioid N-pyrrolidino fluetonitazene in nasal spray**

Fabian Picht ^a, 1,^ *, Valentin Cepus ^b, 2^, Rona Hohlfeld ^b, 3^, Julian Klingbeil ^c, 4^, Marco Weber ^a, 5^

^a^ Institute of Legal Medicine, University of Halle-Wittenberg, Halle (Saale), Germany

^b^ Department of Engineering and Natural Sciences, University of Applied Sciences Merseburg, Merseburg, Germany

^c^ Department of Neurology, University of Halle-Wittenberg, Halle (Saale), Germany

^*^ Corresponding author:

Fabian Picht

University of Halle-Wittenberg

Institute of Legal Medicine

Franzosenweg 1, D-06112 Halle (Saale), Germany

phone: (+49) 345 5571768

fax: (+49) 345 5571587

e-mail: [fabian.picht@uk-halle.de](mailto:fabian.picht@uk-halle.de)

^1^ ORCID: 0009-0000-5641-792X

^2^ ORCID: 0000-0002-5365-9955

^3^ ORCID: 0009-0005-8293-170X

^4^ ORCID: 0000-0002-9306-9212

^5^ ORCID: 0000-0002-6666-0592

# Tables

Table S1. FT-IR data for *N*-pyrrolidino fluetonitazene…………………………………………2

Table S1. FT-IR data for *N*-pyrrolidino fluetonitazene

| Nasal spray | | Reference sample | |
| --- | --- | --- | --- |
| Bands [cm^-1^] | assignment | Bands [cm^-1^] | assignment |
| 3353 | ν(NH) – protonated amine, overlaid with ν(OH) of remnant propylene glycol | 3439 | ν(NH) – protonated amine |
| - | (possibly overlaid by ν(NH)/ ν(OH)) | 3075 | ν(CH) – aromatic, olefinic |
| 2966 | ν(CH) – aliphatic, possibly overlaid by remnant propylene glycol | 2981 | ν(CH) – aliphatic |
| - |  | 1712 | ν(C=O) – citric acid |
| 1617 | ν (C=N) – Schiff base | 1615 | ν (C=N) – Schiff base |
| 1526/1512 | ν_as_(N-O) – nitro group | 1511 | ν_as_(N-O) – nitro group |
| 1452 | ν(C=C) – aromatic | 1449 | ν (C=C) – aromatic |
| 1330 | ν_s_(N-O) – nitro group | 1338 | ν_s_(N-O) – nitro group |
| 1248 | ν(C-F) | 1240 | ν(C-F) |
| 1045 | ν(C-O) – propylene glycol remnant | - | - |
| 819 | δ(CH) - aromatic | 823 | δ(CH) - aromatic |
| 740 | δ(CH) - aromatic | 739 | δ(CH) - aromatic |

# Figures

Figure S1. ^1^H NMR spectrum of reference sample *N*-pyrrolidino fluetonitazene citrate (a) and of nasal spray sample (b) in the range of 0 to 10 ppm chemical shift in DMSO-d6…………………………………………………………...…………………………………..5

Figure S2. ^1^H NMR spectrum of reference sample *N*-pyrrolidino fluetonitazene citrate (a) and of nasal spray sample (b) in the range of 6.5 to 9 ppm chemical shift in DMSO-d6……………………………………...………………………………………………………..6

Figure S3. ^1^H NMR spectrum of reference sample *N*-pyrrolidino fluetonitazene citrate (a) and of nasal spray sample (b) in the range of 1.5 to 5 ppm chemical shift in DMSO-d6………………………………………………………………...……………………………..7

Figure S4. ^1^H NMR spectrum of reference sample *N*-pyrrolidino fluetonitazene citrate (c) and of nasal spray sample (d) in the range of 1.5 to 5 ppm and of reference sample *N*-pyrrolidino fluetonitazene citrate (a) and of nasal spray sample (b) in the range of 6.5 to 9 ppm chemical shift with frequency notations in DMSO-d6………………………………………………………………...……………………………..8

Figure S5. ^13^C NMR spectrum of reference sample *N*-pyrrolidino fluetonitazene citrate (a) and of nasal spray sample (b) in the range of 0 to 200 ppm chemical shift in DMSO-d6……………………………...………………………………………...……………………...9

Figure S6. ^13^C NMR spectrum and DEPT-135 spectrum of reference sample *N*-pyrrolidino fluetonitazene citrate (a) and of nasal spray sample (b) in the range of 0 to 200 ppm chemical shift in DMSO-d6…………...…………………………………………………………………………………10

Figure S7. 2D-HSQC NMR spectrum of reference sample *N*-pyrrolidino fluetonitazene citrate (a) and of nasal spray sample (b) in DMSO-d6.……………………………………………………………………………………………..11

Figure S8. ^1^H-^1^H-COSY NMR spectrum of reference sample *N*-pyrrolidino fluetonitazene citrate (a) and of nasal spray sample (b) in DMSO-d6…………………………………………12


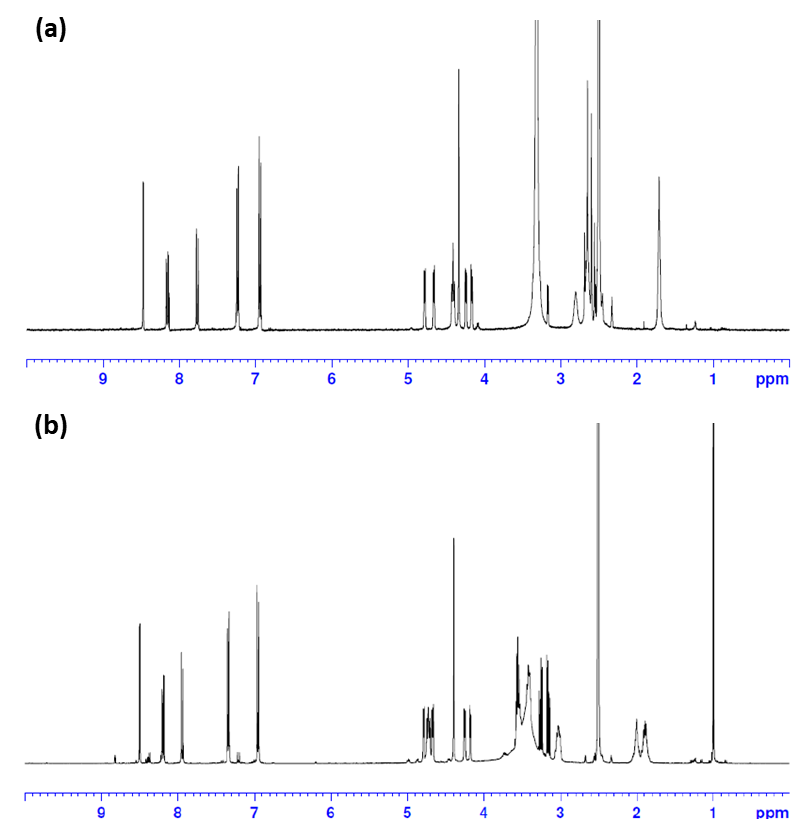


Figure S1. ^1^H NMR spectrum of reference sample *N*-pyrrolidino fluetonitazene citrate (a) and of nasal spray sample (b) in the range of 0 to 10 ppm chemical shift in DMSO-d6


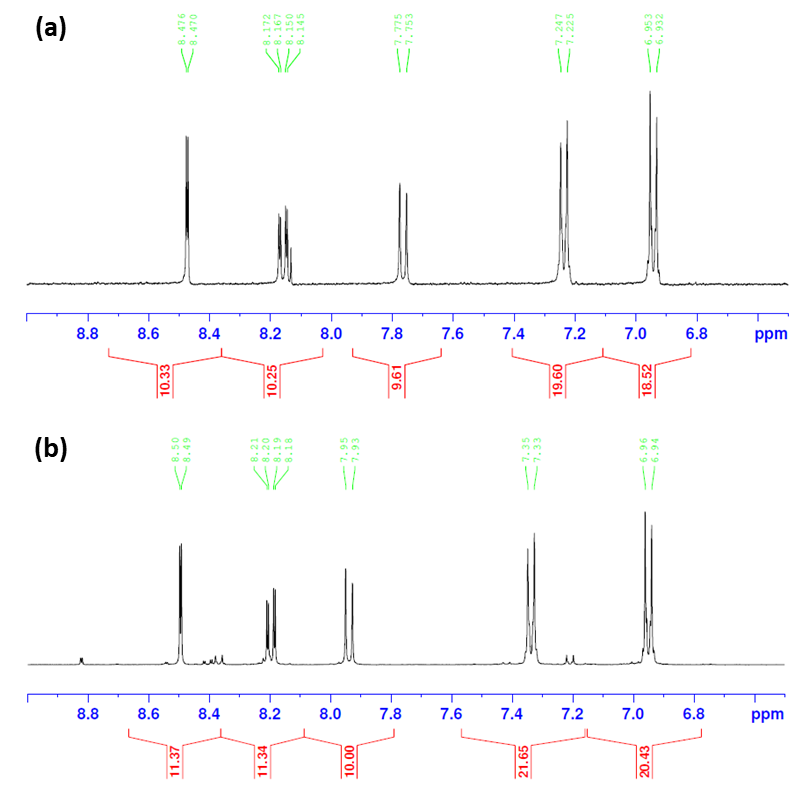


Figure S2. ^1^H NMR spectrum of reference sample *N*-pyrrolidino fluetonitazene citrate (a) and of nasal spray sample (b) in the range of 6.5 to 9 ppm chemical shift in DMSO-d6


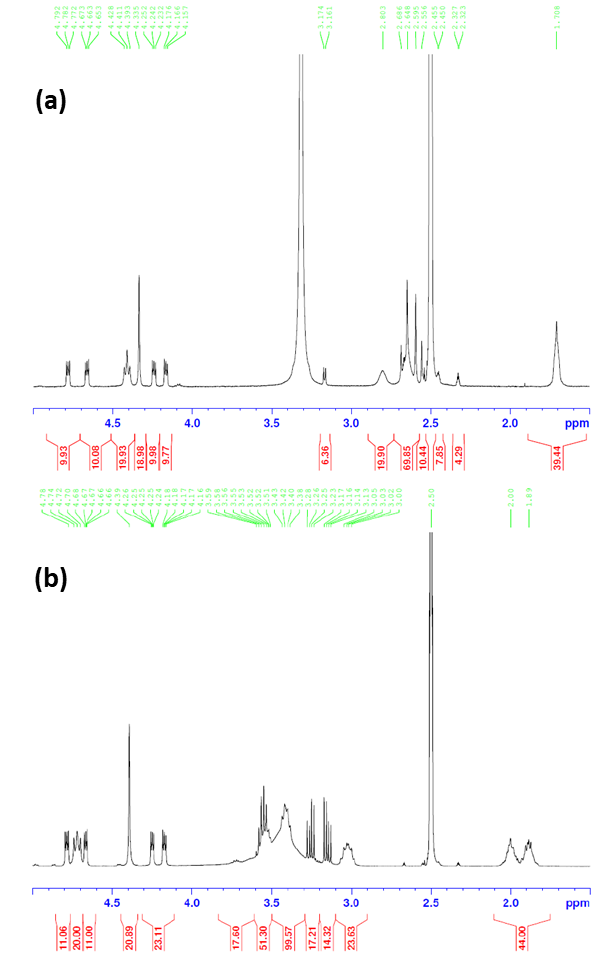


Figure S3. ^1^H NMR spectrum of reference sample *N*-pyrrolidino fluetonitazene citrate (a) and of nasal spray sample (b) in the range of 1.5 to 5 ppm chemical shift in DMSO-d6


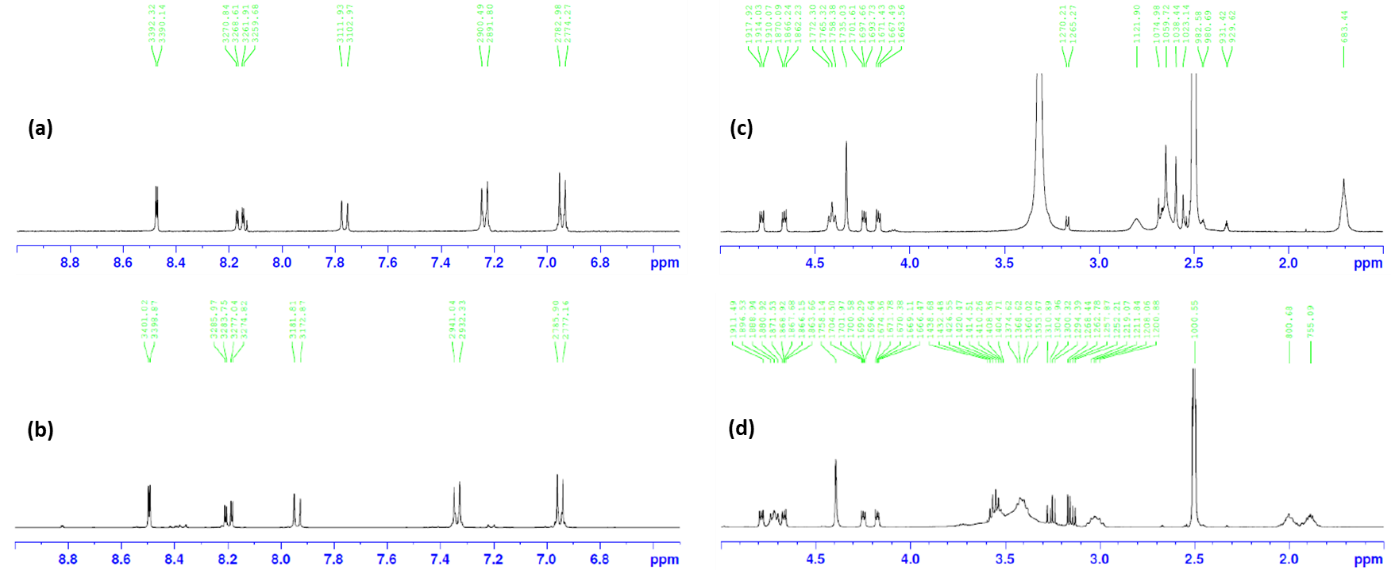


Figure S4. ^1^H NMR spectrum of reference sample *N*-pyrrolidino fluetonitazene citrate (c) and of nasal spray sample (d) in the range of 1.5 to 5 ppm and of reference sample *N*-pyrrolidino fluetonitazene citrate (a) and of nasal spray sample (b) in the range of 6.5 to 9 ppm chemical shift with frequency notations in DMSO-d6


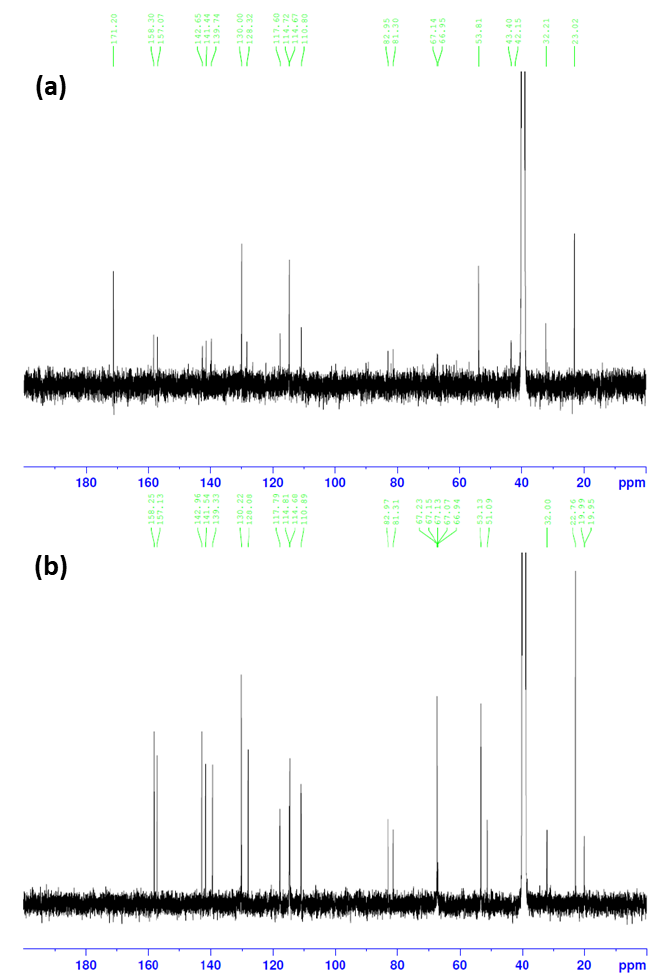


Figure S5. ^13^C NMR spectrum of reference sample *N*-pyrrolidino fluetonitazene citrate (a) and of nasal spray sample (b) in the range of 0 to 200 ppm chemical shift in DMSO-d6


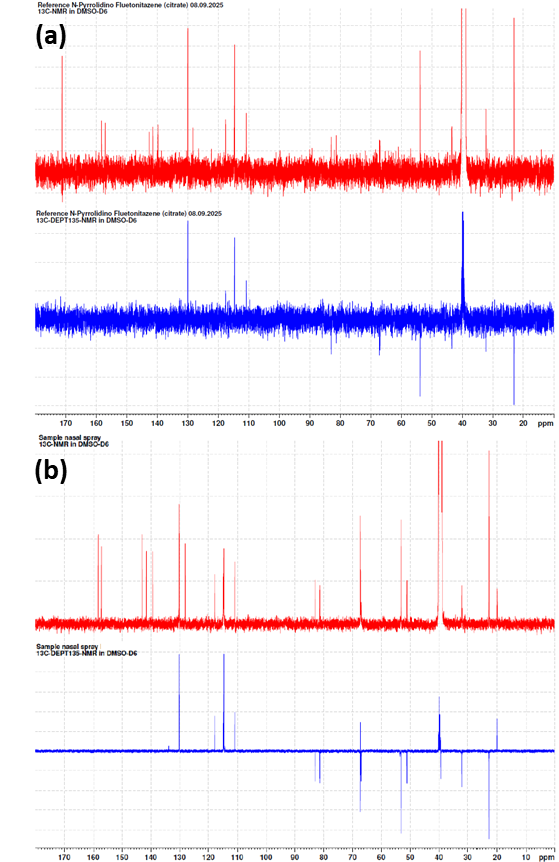


Figure S6. ^13^C NMR spectrum and DEPT-135 spectrum of reference sample *N*-pyrrolidino fluetonitazene citrate (a) and of nasal spray sample (b) in the range of 0 to 200 ppm chemical shift in DMSO-d6


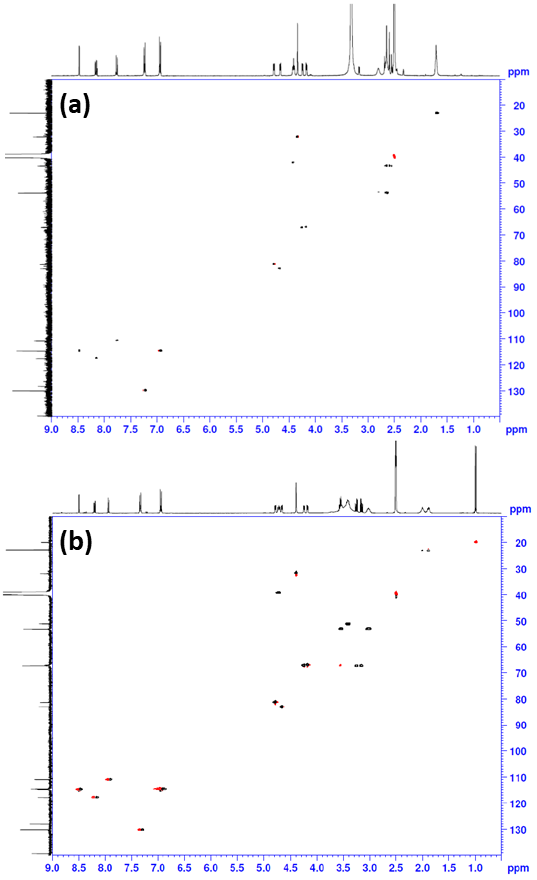


Figure S7. 2D-HSQC NMR spectrum of reference sample *N*-pyrrolidino fluetonitazene citrate (a) and of nasal spray sample (b) in DMSO-d6


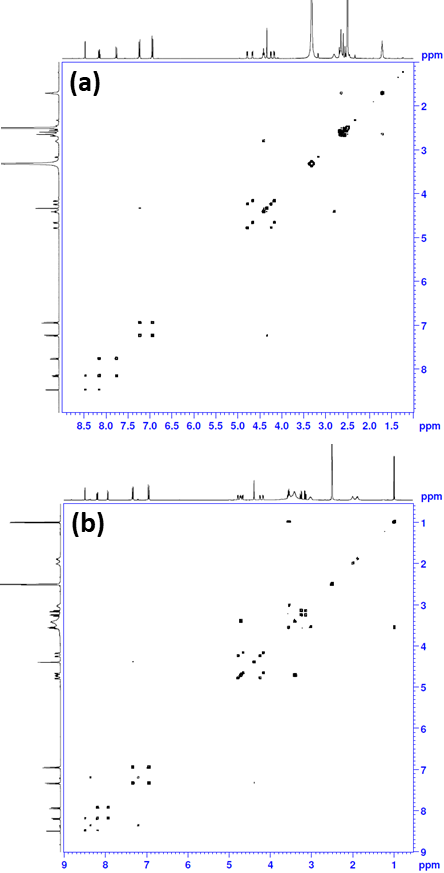


Figure S8. ^1^H-^1^H-COSY NMR spectrum of reference sample *N*-pyrrolidino fluetonitazene citrate (a) and of nasal spray sample (b) in DMSO-d6
